# Keystone Arctic paleoceanographic proxy association with putative methanotrophic bacteria

**DOI:** 10.1038/s41598-018-28871-3

**Published:** 2018-07-13

**Authors:** Joan M. Bernhard, Giuliana Panieri

**Affiliations:** 1 Woods Hole Oceanographic Institution, Department of Geology & Geophysics, MS #52, Woods Hole, MA 02543 USA; 2 CAGE - Centre for Arctic Gas Hydrate, Environment and Climate, Department of Geosciences, UiT the Arctic University in Norway, Dramsveien 201, N-9037 Tromsø, Norway

## Abstract

Foraminifera in sediments exposed to gas-hydrate dissociation are not expected to have cellular adaptations that facilitate inhabitation of chemosynthesis-based ecosystems because, to date, there are no known endemic seep foraminifera. To establish if foraminifera inhabit sediments impacted by gas-hydrate dissociation, we examined the cellular ultrastructure of *Melonis barleeanus* (Williamson, 1858) from the Vestnesa gas hydrate province (Arctic Ocean, west of Svalbard at ~79 °N; ~1200-m depth; n = 4). From sediments with gas hydrate indicators, living *M*. *barleeanus* had unusual pore plugs composed of a thick, fibrous meshwork; mitochondria were concentrated at the cell periphery, under pore plugs. While there was no evidence of endosymbioses with prokaryotes, most *M*. *barleeanus* specimens were associated with what appear to be Type I methanotrophic bacteria. One foraminifer had a particularly large bolus of these microbes concentrated near its aperture. This is the first documented instance of bona fide living *M*. *barleeanus* in gas-hydrate sediments and first documentation of a foraminifer living in close association with putative methanotrophs. Our observations have implications to paleoclimate records utilizing this foundational foraminiferal species.

## Introduction

Methane hydrates are plausible energy sources, but they are also natural hazards because methane decomposition adds carbon to the oceans and atmosphere, contributing to climate change. Furthermore, methane decomposition triggers sediment instability, which can result in submarine landslides and tsunamis. Because hydrate destabilization is caused by warming, recent and anticipated temperature rise is a significant concern, especially in the Arctic where changes are predicted to be earlier and more pronounced compared to lower latitudes^1^. In the Arctic Ocean, methane venting from sediments in shallow water depths (≤~400 m) may be linked to upper-ocean warming^2^; methane release has also been documented from deeper water depths >800 m^3^. Understanding past methane release events, from both deep and shallow waters, is critical for predicting the magnitude and extent of future destabilization events.

Foraminiferal carbonate tests (shells) are crucial repositories encapsulating geochemical proxies instrumental to understanding past oceanographic environmental conditions^4^. The timing and duration of past hydrate dissociation events can be interpreted by analyzing benthic foraminiferal tests from seep areas for their δ^13^C signature^5^. Depletion of δ^13^C (i.e., more negative) in foraminiferal carbonate can indicate of methane release. Benthic foraminiferal tests found adjacent to active methane seeps commonly have depleted δ^13^C values^5–8^. This depletion is interpreted to result from incorporation of DIC (Dissolved Inorganic Carbon), which is even more ^13^C -depleted than the source methane, during the primary biomineralization of the benthic foraminiferal tests, and/or likely ingestion of ^13^C-depleted methanotrophic microbes^6,9^. Furthermore, in both benthic and planktonic foraminifera, extremely negative δ^13^C values (i.e., from −5‰ to −29‰) have been interpreted to reflect precipitation of methane-derived authigenic carbonates as secondary overgrowths on the primary foraminiferal tests^10^. However, even after potential contaminants from authigenic carbonate overgrowths were removed, depleted δ^13^C values remained in many benthic methane-seep foraminiferal tests^8^. The general consensus of many paleoceanographers and paleoclimatologists is that methane emissions are recorded by foraminifera living adjacent to or in active seep areas.

If this truly is the case, a puzzle arises because those foraminifera recording the depleted δ^13^C values include “paleoceanographically relevant” species, which are those commonly used in paleoceanographic reconstructions due to their widespread occurrence in “typical” deep-sea settings, opposed to “extremophile” species. Is it true that these important paleo-proxy species inhabit and calcify at seeps? If so, what allows them physiologically to inhabit these seeps? These paleo-relevant species are not expected to have symbionts or be able to denitrify, like many redoxcline foraminifera^11–15^. If keystone foraminifera have metabolic plasticity, then the paleoceanographic interpretations based on these species need to be seriously reconsidered.

The foraminifer *Melonis barleeanus* is one such keystone for the Arctic that is commonly used in paleoceanographic reconstructions^16–18^. We report here results on the cellular ultrastructure of *M*. *barleeanus* collected from sediments associated with gas hydrate in the Norwegian Arctic.

## Gas Hydrate Emission Site

**Figure 1:**
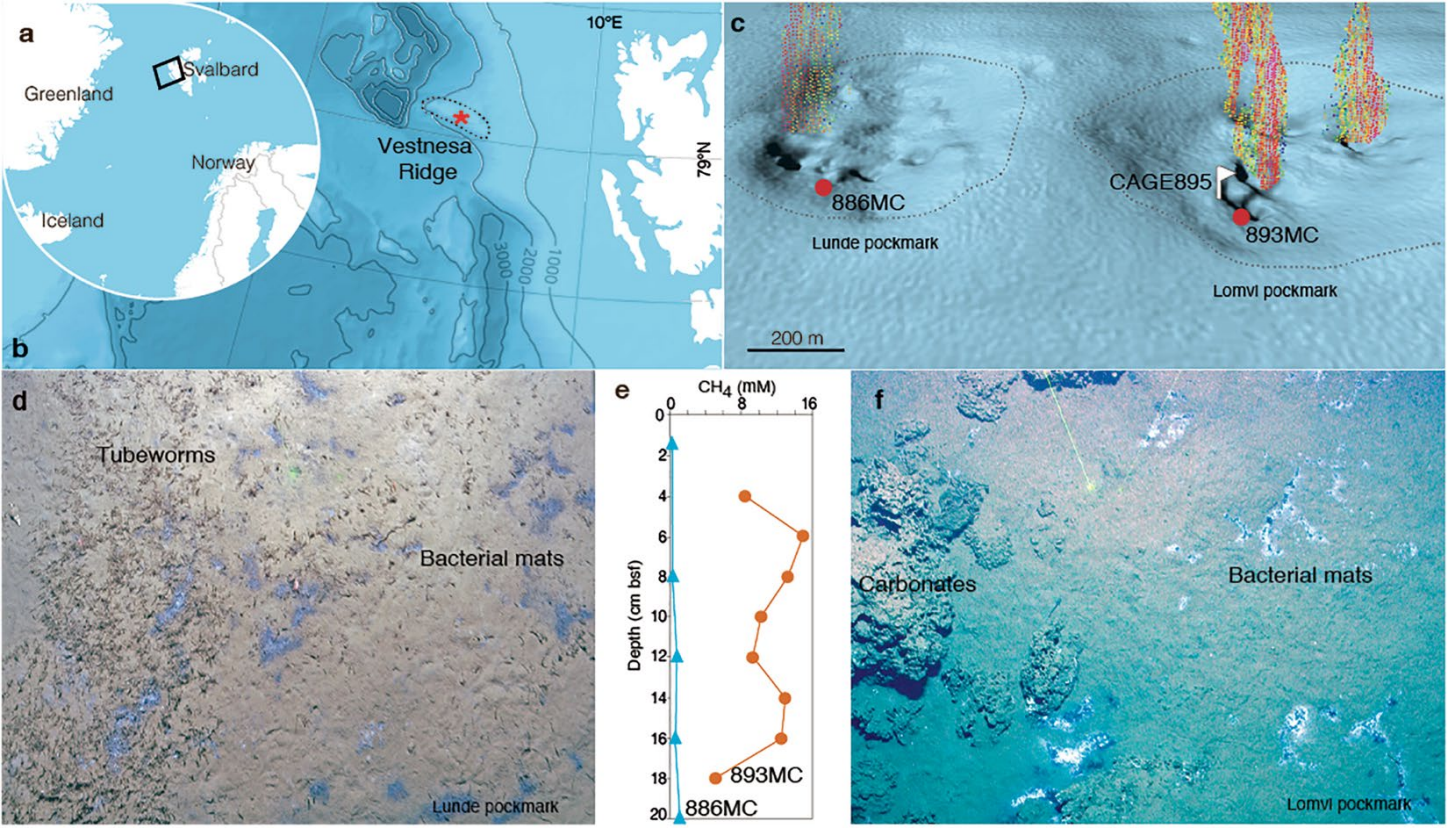
(**a**) Active methane emission sites, Vestnesa Ridge. Map (**b**) is modified from IBCAO^55^. Red * is area shown in c. (**c**) Multibeam bathymetry processed using Kongsberg Neptune software. Gridding and imaging used GMT^56^; visualization made with IVS Fledermaus software. Oblique site view with artist rendition of methane bubbles. Marker CAGE895 notes first sight of near-surface hydrate. Dotted lines delineate pockmarks. (**d**) Seafloor image showing tubeworms and bacterial mats with methane concentrations (**e**) at 886MC. (**f**) Seafloor image showing carbonate and bacterial mats with methane concentrations (**e**) at 893MC. Distance between green laser dots (**d**,**f**) = 20 cm.

The Vestnesa Ridge is a NW-SE trending, ~100-km long, 1–2-km thick contourite located in the Arctic Ocean, west of Svalbard, at ~79°N (Fig. 1a,b). Several depressions in the seafloor, or pockmarks, that are ~700 m in diameter and ~10 m deep relative to the surrounding seafloor are aligned along the ridge summit at water depths of ~1200 m.

Two of the most active Vestnesa Ridge pockmarks are Lomvi and Lunde. They are characterized by low diffuse flow and extensive distribution of bacterial mats and tubeworms over the whole pockmark seafloor. More focused methane outflow emits from ~50-m-diameter depressions called pits (Fig. 1c) that have bacterial mats and carbonate concretions. Emitting from the pits, the methane appears like bubble streams rising into the overlying water column. Pits within the Lomvi pockmark are also characterized by gas hydrate sedimentary layers, each approximately 1–2 cm thick, recovered from sediment cores. The methane emanating from Vestnesa pockmarks has both microbial and thermogenic gas sources^19^.

When methane migrates upward though sediments and encounters sulfate in pore waters, a distinct sediment interval termed the sulfate methane transition zone (SMTZ) forms^20^. In the SMTZ, methane is consumed through anaerobic oxidation of methane (AOM), mediated by a consortium of anaerobic methane-oxidizing archaea (ANMEs) together with sulfate-reducing bacteria^21^ (SRB). ANMEs utilize sulfate as an electronic acceptor, thereby oxidizing carbon in the methane to bicarbonate, favoring the precipitation of methane-derived authigenic carbonates characterized by a δ^13^C value that is strongly depleted^22,23^. The methane that is not consumed in the SMTZ forms gas bubble streams that emerge from small orifices in the seafloor within pits.

Core 15-2-886MC, hereafter referred to as core 886MC, was collected in Lunde pockmark, in a site characterized by diffuse flow, filamentous sulfide-oxidizing bacteria, and chemosynthetic tubeworms (Fig. 1d). The methane concentration was very low in the top 20 cm, only up to 1.2 mM (Fig. 1e). Core 15-2-893MC, hereafter referred to as core 893MC, was collected in an active seep site with focused flow in the Lomvi pockmark characterized by filamentous sulfide-oxidizing bacteria, outcropping carbonate crusts (Fig. 1f), and indurated hydrate crust thought to be sediment cemented by thin gas hydrate layers. The sediment recovered in core 893MC contained laminar (3- to 5-mm thick) and nodular (2–3 cm diameter) gas hydrate features in the upper 10 cm. The vertical methane concentration was relatively high throughout the core (from 7.4 to 13.9 mM in the top 10 cm) along with a slightly concave profile (Fig. 1e). Each of the replicate multicores from 893MC had continuous outgassing in the form of bubbling through small (mm-scale) conduits that occurred for ~30 min upon ascent due to depressurization^19^, confirming the presence of methane in the cores.

Methane emission sites are characterized by oxidation of ^13^C-depleted methane that causes the δ^13^C_DIC_ values to become rapidly depleted within the first few centimeters below the sediment-water interface. The nearby site with diffuse methane flow (886MC), had a δ^13^C_DIC_ of ca −0.54‰ in the surface (top) cm of sediments. On the contrary, in core 893MC, there was a clear decrease in the isotopic values of the dissolved inorganic carbon with increasing sediment depth (i.e., δ^13^C_DIC_ = −3.34‰ at 0–1 cm; −14.25‰ at 1–2 cm; −20.15‰ at 2–3 cm). This vertical pore water δ^13^C_DIC_ gradient indicates that the location of 893MC was geochemically active at the time of collection and that methane oxidation was occurring close to the sediment-water interface. Both our visual observations of the seafloor and geochemical data confirm that the core 893MC collection locality was an active methane emission site when sampled.

## ***Melonis barleeanus*** inhabits methane emission site

**Figure 2:**
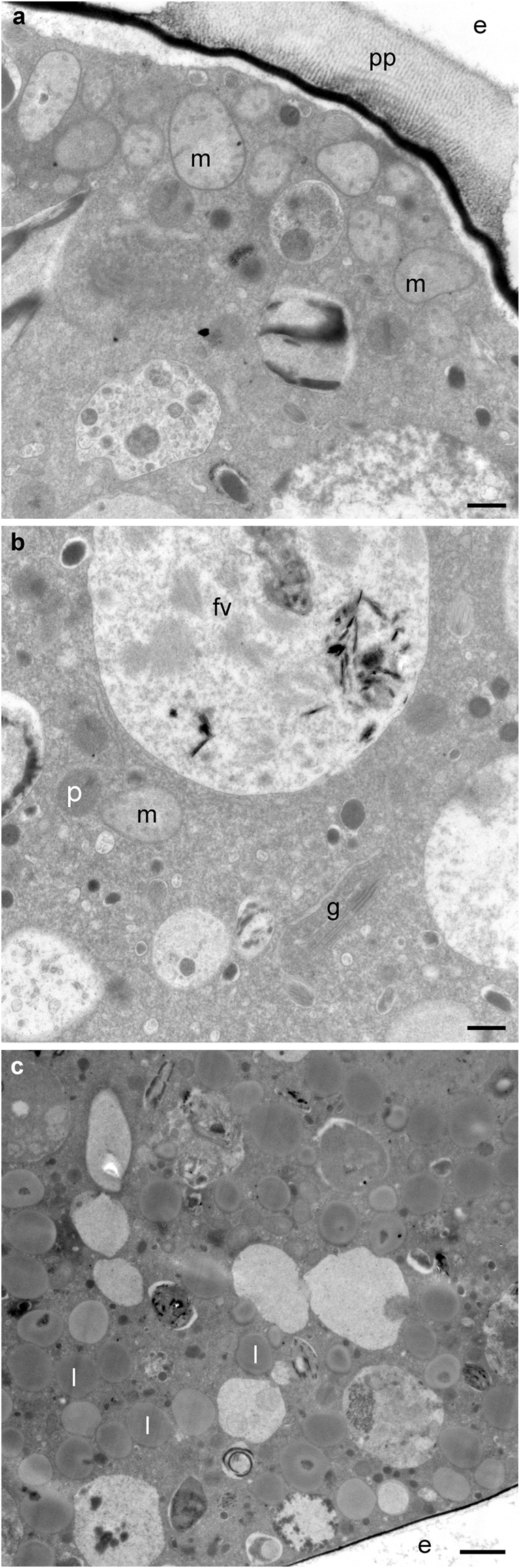
TEM micrographs of observed organelles and other features in *M*. *barleeanus* specimen 4. (**a)** Mitochondria (m) under pore plug (pp) with paracrystalline pattern. (**b**) Cell interior with Golgi (g), mitochondria, peroxisomes (p) and food vacuole (fv). (**c)** Older chamber with numerous lipid reserves (l). e = exterior (environment). Scales: **a**,**b**: 0.5 µm; **c**: 2 µm.

The cellular ultrastructure of all *Melonis barleeanus* examined with transmission electron microscopy (n = 4) had intact organelles of various types, including mitochondria, peroxisomes, food vacuoles, and “empty” vacuoles that are typical of foraminiferal cytoplasm^24^ (Fig. 2; Table 1; Supplementary Figs 1 and 2). Additional organelles such as Golgi apparatus, nuclei, fibrous bodies and lipid droplets were noted in some of the specimens. Because our goal was to assess viability and adaptations, we did not document each organelle type in each foraminiferal specimen. The prevalence of intact mitochondria and at least two other intact organelle types indicates each *M*. *barleeanus* specimen was living at the time of fixation^25,26^.

**Table 1:** List of organelles noted and methanotroph observations for each *M*. *barleeanus* via TEM; m = mitochondria, p = peroxisome, v = vacuole, fv = food vacuole (or residual body), fb = fibrous body, g = Golgi, n = nucleus, l = lipid.

Specimen #	Organelles observed	Methanotroph observations
1	m, p, v, fv, fb, g, n	none
2	m, p, v, fv, fb, l, n	many in apertural region
3	m, p, v, fv, fb, l	many in apertural region + 1 in final chamber
4	m, p, v, fv, fb, g, l	few on exterior (not near aperture)

As noted, *Melonis barleeanus* is commonly used in paleoceanographic reconstructions^8,16–18^. *M*. *barleeanus* has an intermediate to deep infaunal microhabitat^27–29^ thriving on altered organic matter buried in organic-rich silty muddy sediments^29,30^. While some stable isotope analyses of its calcium carbonate test indicate that it favors a rather static position within sediments^31^, some researchers assert that *M*. *barleeanus* has motile behavior, ascending to the sediment-water interface when fresh organics are limited and/or follows bacteria associated with the nitrate-reduction zone^32^. The species and its congener *M*. *pompilioides* have recently been used to provide a low-temperature (i.e., polar and deep sea) paleo-proxy calibration because of their wide ranging distributions geographically and bathymetrically^18^.

Previously, *Melonis barleeanus* has been reported from the Arctic^33^, and found as fossils/relicts in Arctic gas hydrate areas^8,10,16^. The species has also been associated with hydrate pockmarks in lower latitude deep-water sites^34^. In the Arctic, *M*. *barleeanus* can indicate high sedimentation rates with low and steady food supply, and it has been associated with the presence of Atlantic-derived water^33,35^. The congener *Melonis zaandami* has also been reported from Arctic sediments^36^. Furthermore, *M*. *zaandami* collected from Håkon Mosby Mud Volcano (Arctic) grew in high-pressure methane-enriched experiments^37^. *M*. *zaandami* have moderately depleted δ^13^C signatures (−2.40 ± 0.07‰), from the region off Svalbard^36^.

**Figure 3:**
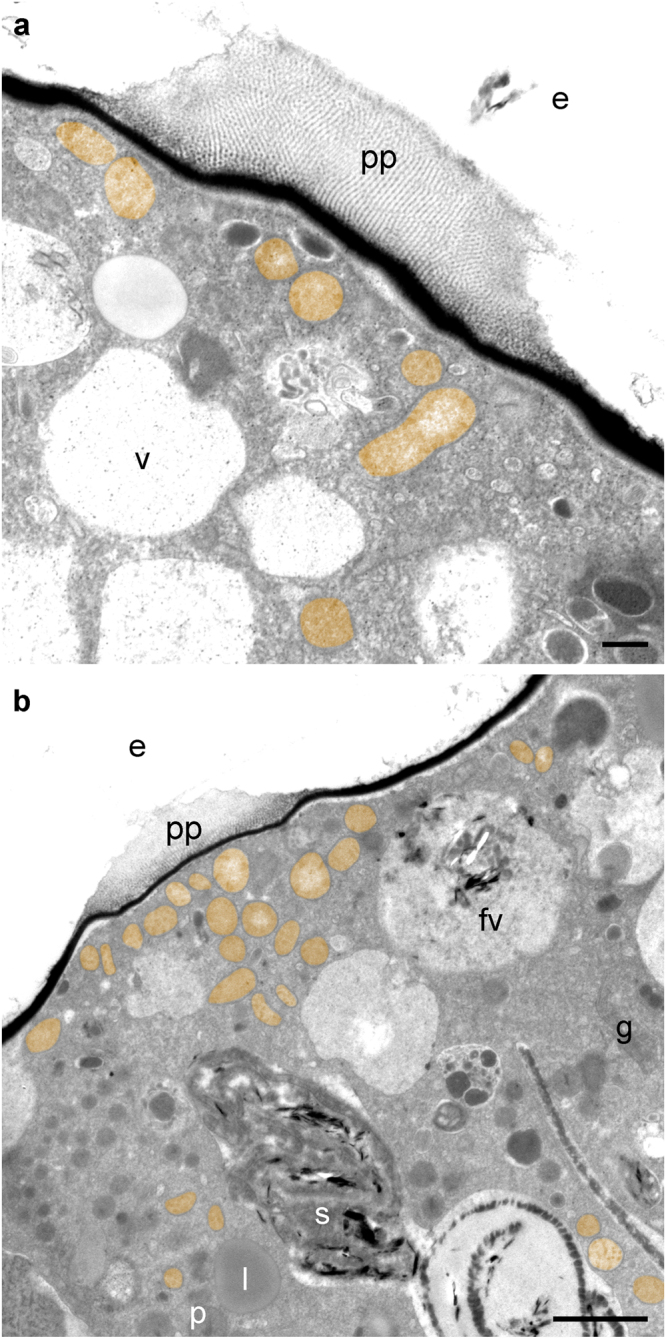
TEM micrographs of pore plug regions in *M*. *barleeanus*. (**a**) Mitochondria (colored orange) under a pore plug of specimen 1. (**b**) Mitochondria (orange) appearing more concentrated under pore plug compared to endoplasm, in specimen 4. Key to features is the same as in Fig. 2 caption; s = stercomata-like feature. Scales: **a**: 0.5 µm; **b**: 2 µm.

The *Melonis barleeanus* mitochondria were typically more abundant under pores compared to the interior cytoplasm or non-pore cell periphery (Figs 2a and 3; Supplementary Fig. 2a,c and d). Such occurrences are consistent with mitochondrial distributions in specimens of many other foraminiferal species from chemocline habitats^14,24,38^. Assuming the *M*. *barleeanus* mitochondria use oxygen such dispersion suggests optimization of oxygen acquisition by association with these openings in the calcitic test. Even if another electron acceptor is used by the mitochondria (e.g., hydrogen sulfide)^39^, their distribution in *M*. *barleeanus* implies scarcity of the electron acceptor.

The test pores of *Melonis barleeanus* were unusual in being filled with paracrystalline material (Figs 2a and 3) underlain by a relatively conventional electron-opaque inner organic lining (Figs 2a,c and 3; Supplementary Fig. 2). All four specimens had these paracrystalline pore plugs. The banding pattern of this paracrystalline material appears similar to that of the atypical tubulin polymorph characteristic of foraminifera^40^. The significance of this paracrystalline material is currently unknown. Specimens of another foraminiferan inhabiting a redoxcline have modified pore plugs where plasma membrane invaginations extend into the cytoplasm toward mitochondria clustered beneath the pores^14^; no such invaginations were observed, however, in *M*. *barleeanus*. As noted, the apparent association of mitochondria and pores in *M*. *barleeanus* suggests that oxygen or another electron acceptor passes through these pores; further dedicated study is required to confirm this inference and its implications.

Peroxisomes in *Melonis barleeanus* occurred in small clusters of ~3–10 (Supplementary Figs 1a and 2c) rather than as singlets, which are typical of benthic foraminifera from aerated deep-sea habitats^41^. The *M*. *barleeanus* peroxisome clusters were not as extensive as those peroxisome fields complexed with endoplasmic reticulum documented from other chemocline foraminifera such as *Nonionella stella* and *Buliminella tenuata*^42^, where congregations can have many dozens to hundreds of peroxisomes. This lack of peroxisome proliferation suggests that these *M*. *barleeanus* specimens were not under significant oxidative stress and/or that concentrations of reactive oxygen species (e.g., hydrogen peroxide) were not high in these sediments.

While a sample size of four may be considered low, we analysed all available *M*. *barleeanus* present in available glutaraldehyde-fixed material. There were no cytoplasm-bearing *Melonis barleeanus* in the glutaraldehyde-preserved material from the surface cm of 886MC. Thus, *M*. *barleeanus* ultrastructural observations from that site were not possible.

## Methanotroph-like associates of ***Melonis barleeanus***

**Figure 4:**
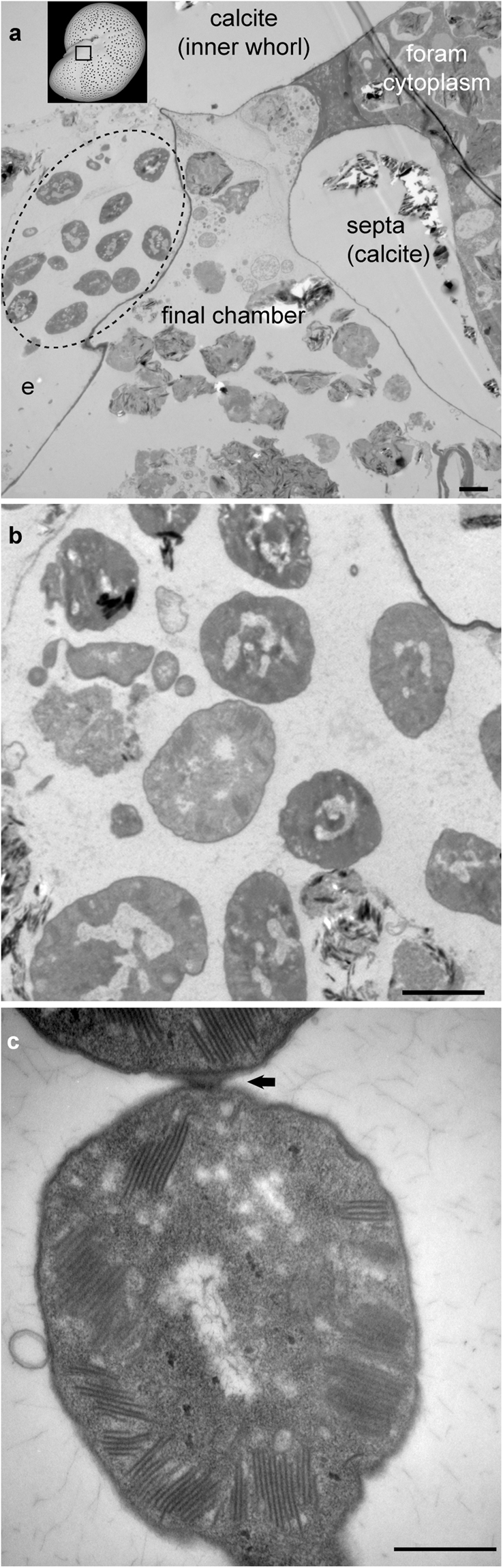
TEM micrographs of microbes associated with *M*. *barleeanus* (specimen 3). (**a**) Overview image showing part of final and penultimate foraminiferal chambers, with microbes in apertural region (outlined by dotted ellipse). e = exterior (environment). Inset, Scanning electron micrograph of *M*. *barleeanus* where black box delimits area appearing in panel a. (**b**) Image from another section of the same *M*. *barleeanus* demonstrating the cluster of microbes extends through much of apertural region. (**c**) Higher magnification view of microbes (from **a**), showing stacked intracytoplasmic membranes (linear features); note attachment (arrow) between two microbes. Scales: (**a**,**b)** 2 µm; (**c)** 0.5 µm.

Numerous microbes of one morphotype existed in the apertural region of two *Melonis barleeanus* specimens (specimens #2, #3; Fig. 4a,b; Supplementary Fig. 3). The apertural region is where the final (most recently formed) test (shell) chamber opens into the environment, providing a place for the foraminiferal reticulopods to exit and re-enter the test. Thus, this region is the transition between environment and test interior. Because reticulopods are so dynamic, determining the exact boundary of the foraminiferal cell in this region is difficult.

These microbes had numerous intracellular stacked membranes (Fig. 4c; Supplementary Fig. 3a,b). A subset of these microbial cells was attached in pairs (Fig. 4a,c; Supplementary Fig. 3a,b). A third foraminiferal specimen (#4) had similar microbes, although these were not in the apertural region, but loosely affiliated with the foraminifer’s exterior, removed from the aperture. One *M*. *barleeanus* specimen lacked similar microbes, but because a full serial section was not performed on this specimen, it is possible that similar microbes were present but undetected.

In one instance, a similar microbe with stacked membranes occurred in the first chamber of *Melonis barleeanus* specimen #3 (Supplementary Fig. 4), indicating active uptake by the foraminifer. Well established instances of foraminiferal (non-photosynthetic) endosymbiosis reveal abundant and obvious endobionts^15,43^. There was no evidence of abundant endobionts of any morphology in the *M*. *barleeanus* cytoplasm, there is no evidence of endosymbiosis in Lomvi *M*. *barleeanus*.

Morphologically, these foraminiferal-associated microbes appear similar to known Type I methanotrophic bacteria, which also have stacked intracytoplasmic membranes^44,45^. Type I methanotrophs oxidize methane aerobically. An association between foraminifera and methanotrophic bacteria could impart a depleted δ^13^C value, as has been noted in other seep-related fauna^46,47^.

Rarely, additional microbial morphotypes occurred as individuals among inorganic debris associated with the test exterior of each *M*. *barleeanus*. These microbes did not have stacked membranes and were varied in shape and size (Supplementary Fig. 5); all of these microbes were sparsely distributed.

Because methanotrophic bacteria commonly occur at methane emission sites, it may not be surprising that the *M*. *barleeanus* living at this site are associated with methanotroph-like microbes. Association of methanotrophs and metazoans is common at seeps and vents, especially in the form of symbiosis^48^. Thus, while the association of *M*. *barleeanus* and the methanotrophs may be circumstantial, it is also plausible that the association may be a type of symbiosis. Symbiosis is a close association involving biological interaction between two or more species. Symbioses between foraminifera and microbes are not uncommon, especially among planktonic and reef foraminifera, which typically have photosynthetic symbionts^49^. Many benthic foraminifera also have symbionts, including non-photosynthetic types^50^. Some foraminiferal species have facultative symbioses, with variability of microbial-associate presence, where some foraminiferal populations have symbionts while other populations or individuals lack such associations, e.g., *Buliminella tenuata*^50^. Such associations could be considered a transitional symbiosis, between permanent symbiosis (i.e., mutualism or commensalism) and parasitism^25,50^. Until we have data from more populations, we consider the association between Lomvi *M*. *barleeanus* and these methanotroph-like microbes to be a type of putative symbiosis.

While it is possible that *Melonis barleeanus* harbour endosymbiotic methanotrophs, to date, evidence for this has not been observed. Examination of conspecifics from additional gas hydrate emission sites will help resolve this conundrum regarding *M*. *barleeanus* specifically, benthic foraminiferal symbioses in general, as well as seep-associated foraminiferal carbonate δ^13^C disequilibrium, if foraminifera precipitate calcite while living in such habitats. While some studies^51,52^ conclude foraminifera do not grow during active seepage, others show evidence of foraminiferal growth in seeps (via isotopic signatures^25^).

In conclusion, this is the first documented association of a foraminifer and putative methanotrophic bacteria. Given that *Melonis barleeanus* is used for paleoceanographic reconstructions, knowledge of their association with methane-oxidizing bacteria is imperative to enable accurate environmental assessments. Further dedicated studies will determine if the methanotrophs impact *M*. *barleeanus* carbonate isotope values.

## Methods

### Sampling

Samples were collected in May 2015 aboard the R/V *Helmer Hanssen* during CAGE 15–2 cruise from the Lomvi and Lunde pockmarks, from a water depth of ~1200 m, via a combined *TowCam-Multicorer* system (TC-MC) that allowed for the collection of a maximum of six ~60-cm-long, real-time visually-guided cores (MISO; http://www.whoi.edu/website/miso). Of the replicates from each multicorer recovery, one was used for porewater and micropalentological sampling, and an adjacent core in the TC-MC frame was used for headspace gas analyses. Core 15-2-893MC (79.0030°N, 06.9239°E), referred to as core 893MC, and core 15-2-886MC (79.0061°N, 06.9005°E), referred to as core 886MC, were collected on 21 May 2015 from 1203-m and 1209-m water depths, respectively. Core 893MC site was characterized by filamentous sulfide-oxidizing bacteria and outcropping carbonate crusts whereas 886MC site by filamentous sulfide-oxidizing and tubeworms. Upon recovery, multicore sediments were sectioned into one-centimeter intervals to a depth of 3 cm. Multicore intervals were subsampled for multiple types of analyses, as described below.

### Methane concentrations

Interstitial gas from sediments in multicores MC893 and MC886 was sampled at different intervals (Fig. 1e) using a conventional headspace technique^53^. Gas analyses were performed with a ThermoScientific Trace 1310 gas chromatograph equipped with a ThermoScientific TG-Bond Alumina (30 m × 0,53 mm × 10 μm column) and a flame-ionization detector (GC-FID).

### DIC isotopic analyses

Sediments were sampled for porewater DIC (Dissolved Inorganic Carbon) analyses using rhizons every cm for the top 3 cm. In the 1–2-cm and 2–3-cm intervals of 886MC, there was insufficient porewater recovery to allow analyses. Porewater sampling was done following the method described in previous publications^54^, where aliquots of water were poisoned onboard with HgCl_2_ for the measurement of DIC. δ^13^C analyses of DIC (±0.04‰ precision) were performed using a Delta V mass spectrometer coupled to a Finnigan Gasbench at Oregon State University.

### Transmission Electron Microscopy

For foraminiferal ultrastructural studies, as soon as possible after multicore recovery aboard the surface vessel (within ~30 minutes), sediment-sample aliquots were preserved in chilled 4% TEM-grade glutaraldehyde in 0.1 M cacodylic acid-sodium salt buffer, following standard protocol^13^. Samples were kept cold during transport to the shore-based laboratory.

In the laboratory, a saturated solution of Rose Bengal was introduced to glutaraldehyde-preserved sediment samples for at least 24 hours. Samples were washed with buffer over a 63-µm screen to remove fixative and stain. The >63-µm fraction was examined with a stereo-dissecting microscope. Rose-Bengal-stained individuals were readily identifiable and isolated from the 0–1 cm section of 893MC. All Rose-Bengal stained *Melonis barleeanus* specimens (n = 4) were prepared for TEM using Bernhard’s standard methods^13^; thin sections were examined on a JEOL JEM-200CX TEM operated at 100 KV.

### Data availability

Additional TEM images are available from the corresponding author (JMB) upon reasonable request. Cruise and sample information, seafloor images, and geochemistry data are available from Giuliana Panieri upon reasonable request.

## Electronic supplementary material

Supplementary figures
