# Supplementary material for: Keystone Arctic paleoceanographic proxy association with putative methanotrophic bacteria
Source: Sci Rep. 2018 Jul 13;8:10610. doi: 10.1038/s41598-018-28871-3 (PMC6045607; doi:10.1038/s41598-018-28871-3)
Supplement: Supplementary file 1 — Supplementary figures [file 41598_2018_28871_MOESM1_ESM.pdf]

Keystone Arctic paleoceanographic proxy association with putative methanotrophic bacteria

Joan M. Bernhard[1*#] and Giuliana Panieri[2#]

[1]Woods Hole Oceanographic Institution, Department of Geology & Geophysics, MS #52, Woods Hole, MA 02543, USA

[2]CAGE - Centre for Arctic Gas Hydrate, Environment and Climate, Department of Geosciences, UiT the Arctic University in Norway, Dramsveien 201, N-9037 Tromsø, Norway

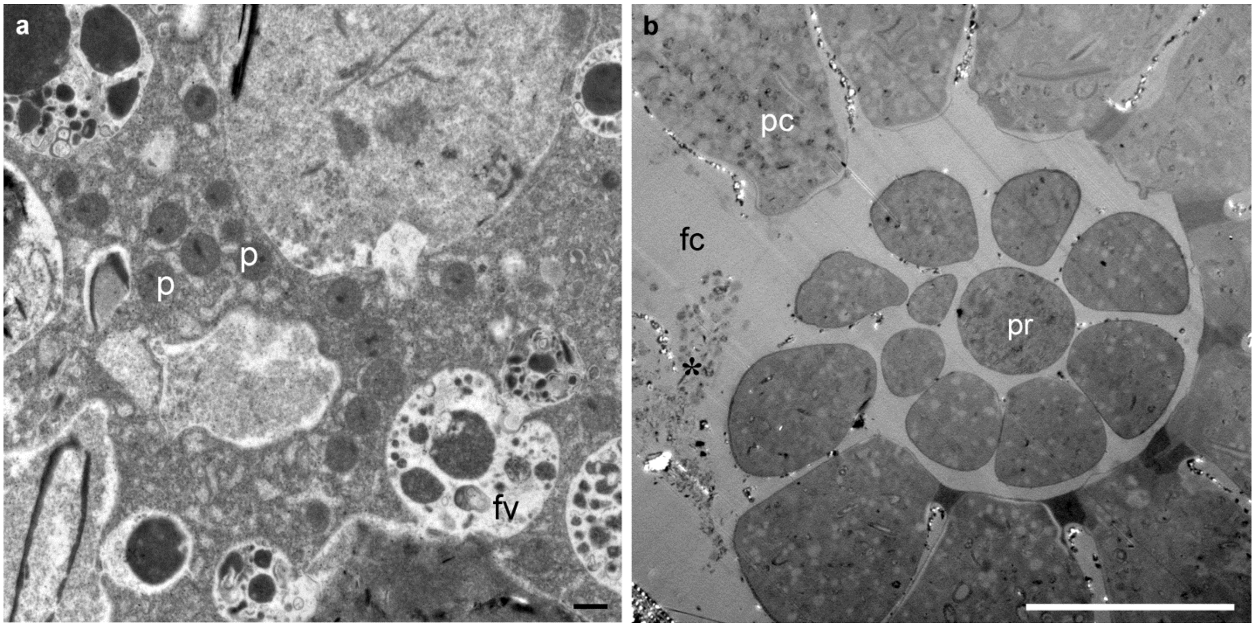

Supplementary Figure 1. TEM micrographs of *M. barleeanus*. (**a**) Cytoplasm of specimen 3 showing peroxisomes (p) and food vacuoles (fv). (**b**) Low magnification view of through entire whorl of specimen 4, showing material and debris associated with apertural region (*). fc = final chamber; pc = penultimate chamber; pr = proloculus. Scales: a = 0.5 μm; b = 100 μm.

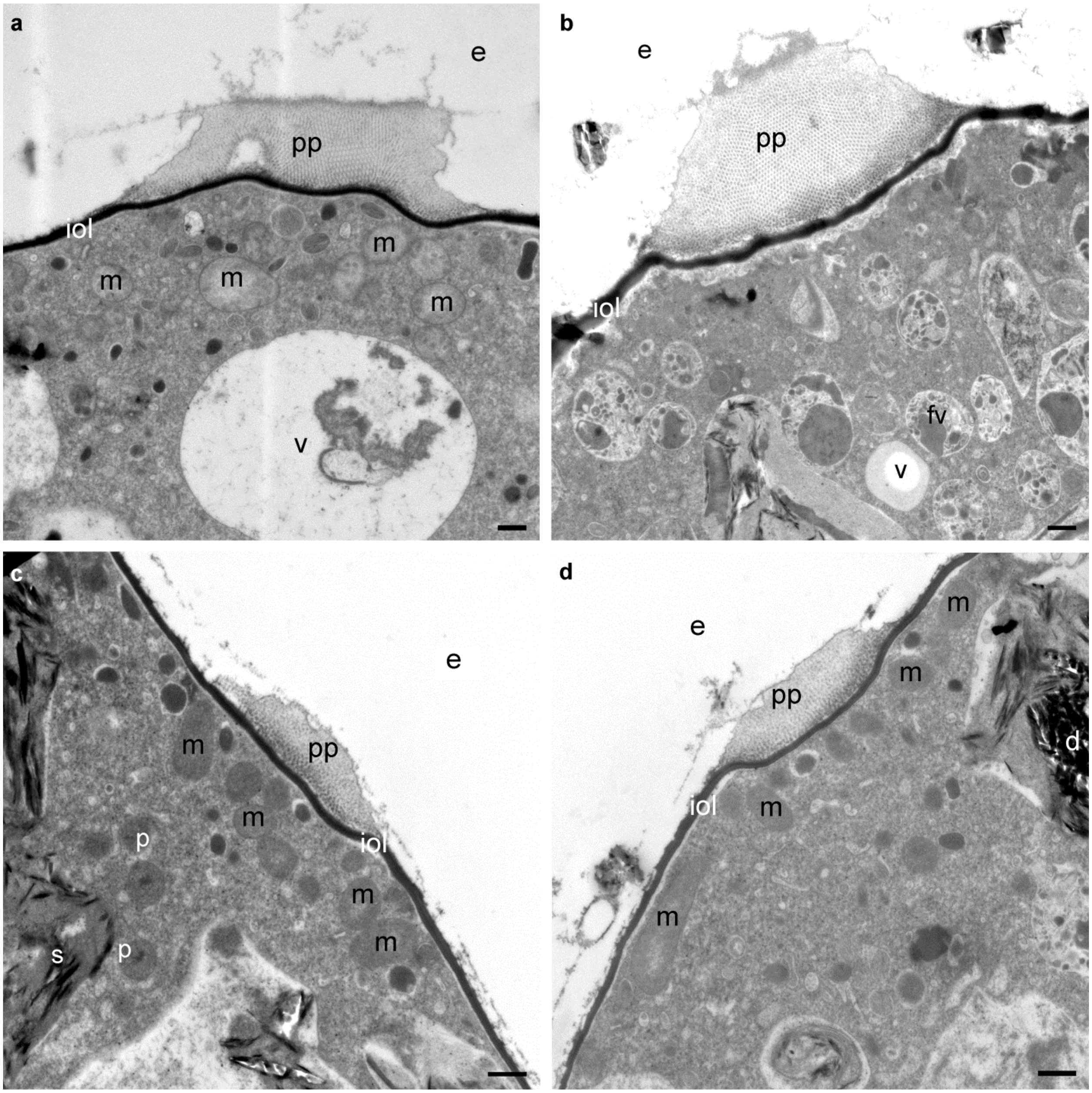

Supplementary Figure 2. TEM micrographs of *M. barleeanus* showing cross sections of pore plugs and underlying cytoplasm. (**a**) Note mitochondria are concentrated at the cell periphery (specimen 4). (**b**) Pore plug of specimen 2 lacking obvious mitochondria. Because the cytoplasm is so dense, mitochondria may be present but not discernable. (**c, d**) Mitochondria concentrated under pores of specimen 3. pp = pore plug; iol = inner organic lining; m = mitochondria; v = vacuole; fv = food vacuole; p = peroxisomes; d = inorganic debris; s = stercomata-like feature. Scales: a-d = 0.5 μm.

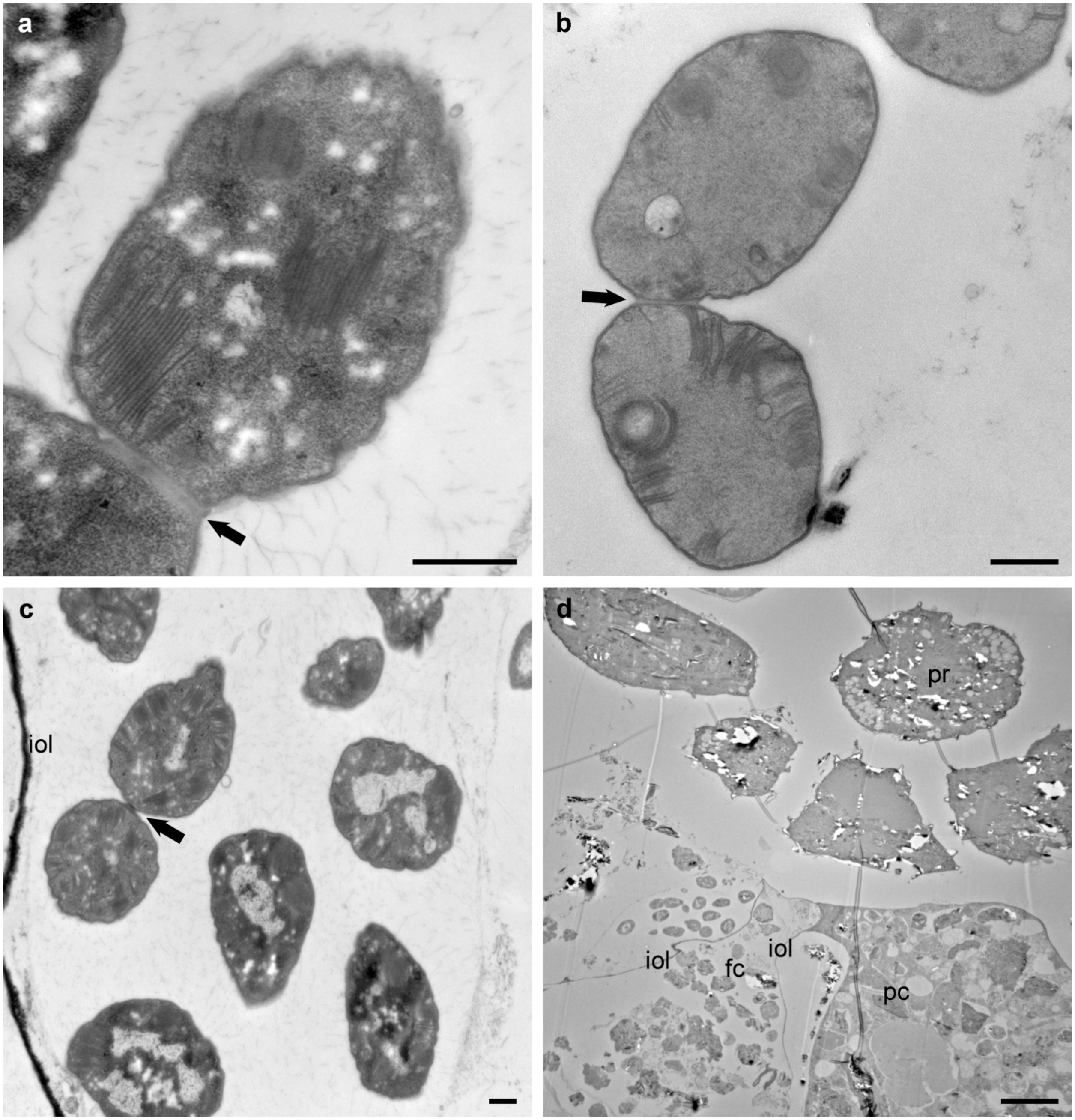

Supplementary Figure 3. TEM micrographs of microbes associated with *M. barleeanus*. (**a**) Close up showing stacked membranes in microbe attached (arrow) to a similar cell, on exterior of specimen 3. (**b**) A pair of microbes with stacked membranes associated with specimen 2. (**c**) Cluster of microbes in apertural region of specimen 3. This is a subset of microbes shown in Fig. 4a. (**d**) View of specimen 3 showing at low magnification, the orientation of microbe cluster in the apertural region. iol = inner organic lining; pr = proloculus. Scales: a-c = 0.5 μm; d = 10 μm.

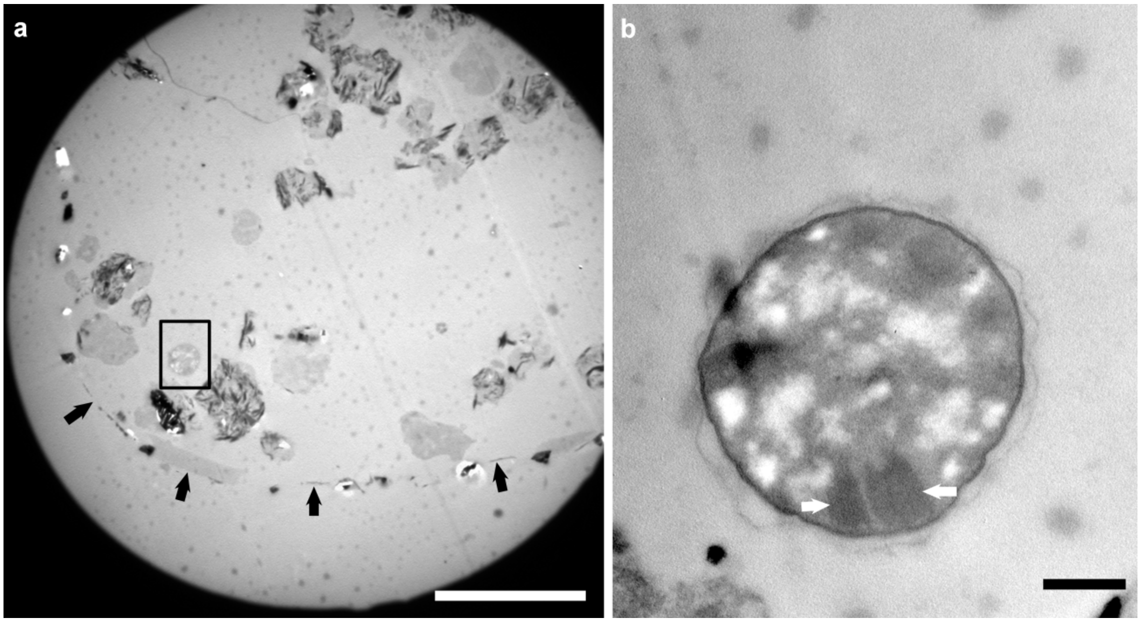

Supplementary Figure 4. TEM micrographs of *M. barleeanus* (specimen #3). (**a**) Low magnification view showing final chamber (delimited by black arrows), containing what could be a Type I methanotroph (outlined box). (**b**) Higher magnification view showing possible Type I methanotroph, where stacked membranes are discernable (white arrows). Scales: a = 10 µm; b = 0.5 µm.

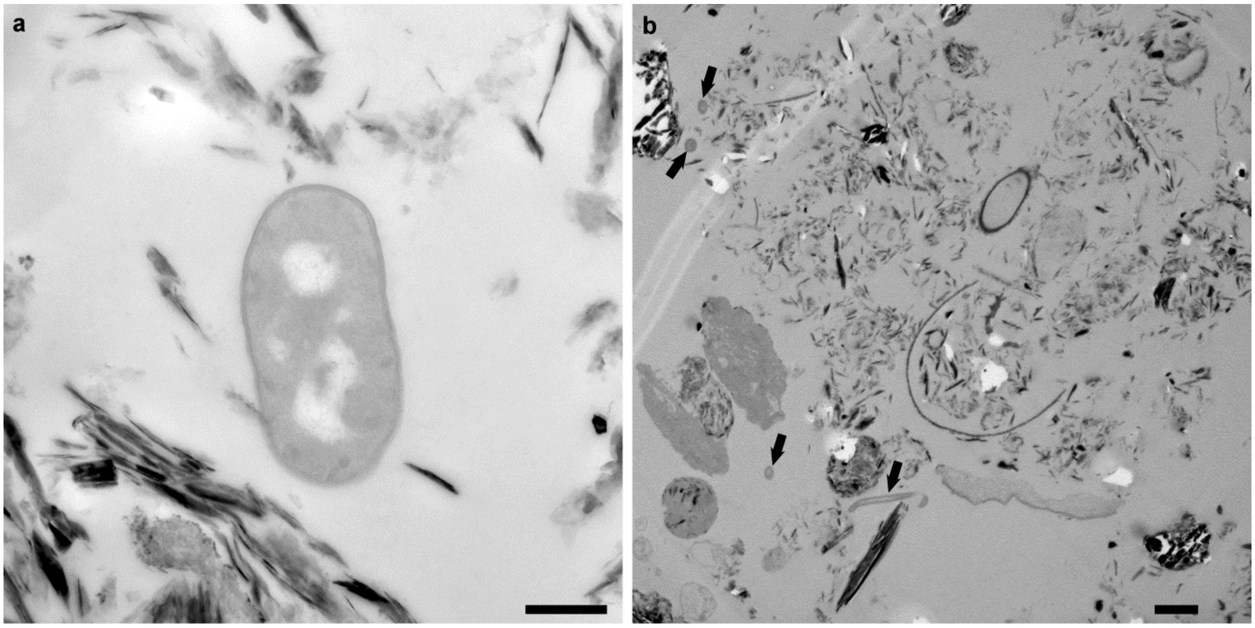

Supplementary Figure 5. TEM micrographs of *M. barleeanus*. (**a**) rod-shaped microbe associated with exterior of specimen 1. (**b**) Low magnification view of exterior-associated materials on specimen 4 showing microbes of varied size and shape (black arrows). Scales: a = 0.5 μm; b = 2 μm.
